# MiR-124 Negatively Regulated PARP1 to Alleviate Renal Ischemia-reperfusion Injury by Inhibiting TNFα/RIP1/RIP3 Pathway

**DOI:** 10.7150/ijbs.58163

**Published:** 2021-05-13

**Authors:** Jing Ke, Fan Zhao, Yanwen Luo, Fangjing Deng, Xiongfei Wu

**Affiliations:** 1 Department of Nephrology, Renmin Hospital of Wuhan University, Wuhan, China.; 2 Department of Endocrinology, Ezhou Central Hospital, Ezhou, Hubei, China.

**Keywords:** MiR-124, PARP1, TNFα/RIP1/RIP3 pathway, renal ischemia-reperfusion injury.

## Abstract

Renal ischemia-reperfusion injury (IRI) is one of the underlying causes of acute kidney injury and also an unavoidable problem in renal transplantation. Lots of miRNAs and targets have been found to participate in some post-transcriptional processes in renal IRI, however, the detailed knowledge of miRNA targets and mechanism is unknown. In this study, miR-124 was found inhibited and PARP1 was overexpressed in renal IRI cells and mouse models. Dual-luciferase reporter assay revealed that miR-124 post-transcriptionally regulated PAPR1 3′UTR activity. Our results also demonstrated miR-124 negatively regulated PARP1 which played a role in necroptosis of renal ischemia-reperfusion injury by activating TNFα. TNFα induced the RIP1/RIP3 necroptosis signaling pathway to aggravate the renal injury. Collectively, these studies identified PARP1 as a direct target of miR-124 and activated RIP1/RIP3 necroptosis signaling pathway through TNFα. It elucidated the protective effect of miR-124 in renal ischemia-reperfusion injury, which demonstrated the regulatory mechanism of miR-124/PARP1 in renal injury and exhibited the potential as a novel therapeutic for the treatment of renal IRI.

## Introduction

Ischemia-reperfusion injury (IRI) refers to the phenomenon that the damages of the ischemic tissue and organ are aggravated after the resumes of blood supply. The kidney has a special physiological structure and function, which is especially predisposed to be affected by ischemia and hypoxia, so the patients who occur renal IRI are at greater risk of acute renal injury (AKI) and high mortality 1. Moreover, renal IRI is an unavoidable problem in renal transplantation 2 and the pathogenic mechanism underlying renal IRI is complicated. Several factors including oxidative stress 3, mitochondrial dysfunction 4, energy metabolism 5, cell necrosis and apoptosis 6, and inflammation 7 are all attributed to renal IRI injury. Therefore, exploring the molecular regulation mechanism of renal IRI occurrence and development is not only of great importance in finding effective strategies to prevent renal IRI and AKI, but also is of major significance to improve the prognosis of renal transplantation.

MicroRNAs are highly conserved small RNA molecules of 21-25 nucleotides, which have been reported to be associated with renal IRI 8. For example, miR-687 was markedly upregulated during renal ischemia-reperfusion injury in mice and cell models 9. Another data showed that miR-21 was suppressed in bone marrow-derived dendritic cells (DCs), mice with miR-21 deficiency in DCs subjected to renal IRI showed more severe renal dysfunction and inflammatory response compared with wild-type mice^10^. Besides, miR-127 was also found to be increased to protect proximal tubule cells against renal IRI 11. Although some miRNAs have been found the protective effects on renal IRI, the protected mechanism is still not well understood. Emergent data showed miR-124 is decreased expression and MMP2 as a direct target of miR-124 in renal epithelial cells when suffered hypoxia 12. Overexpressed miR-124 effectively inhibited chronic inflammation in renal tubular epithelial cells induced by LPS 13. Low expression of miR-124 was detected in renal cancer cell lines OS-RC-2, 769-P and HK-2 cells, and miR-124 inhibited the invasion of cancer cells 14. However, the mechanism of miR-124 in renal IRI is not clear and further research is needed to functionally elucidate.

The necroptosis complex is mainly composed of RIP1, RIP3 and MLKL, which was previously believed to be the main form of cell death after ischemia-reperfusion injury 15. Recent studies have found that Nec-1, an inhibitor of RIP1, protected C57BL/6 mice against renal ischemia-reperfusion injury 16. Besides, knockout RIP3 improved renal function and renal injury in mice 17. RIP1/RIP3 pathway was involved in the regulation of renal ischemia-reperfusion injury through cell necroptosis 18. Additionally, PARP1 inhibitor or knockout of PARP1 can alleviate renal ischemia-reperfusion injury and protect the kidney 19. Thus, it can be seen that PARP1 regulates the renal IRI through a variety of pathways based on the poly-ADP-ribose (PAR) modification activity and protein level 20. Recent data identified a highly conserved binding site of miR-124 in the 3′UTR of PARP1, miR-124 post-transcriptionally regulated PARP1 activity in a dopaminergic neuronal cell model 21, However, the research about the mechanism and the relationship of miR-124 and PARP1 in the process of renal IRI is extremely limited.

In our study, we investigated the regulatory mechanisms of miR-124, the deteriorated effect of PARP1, and the relationship between them in renal IRI both *in vivo* and *in vitro*. Our finding revealed a new direction and theoretical basis for renal IRI protection, and also provided clues for the development of new strategies of AKI and renal transplantation.

## Methods

### Cell culture, model, and transfection

Human renal tubular epithelial cell line HK-2 and Rat proximal tubule cell line RPTCs were cultivated in Dulbecco's modified Eagle's medium/F12 (Hyclone, Rockville, MD) containing 10% fetal bovine serum, penicillin (10 units/μl), and streptomycin (10 units/ml) (Gibco, Carlsbad, CA) at 37°C in an incubator (Thermo Fisher Scientific, Marietta, OH). To construct the HK-2 and RPTCs renal IRI model, cells were cultured with serum-free medium and exposed to hypoxia in a tri-gas incubator (94% N_2_, 5% CO_2,_ and 1% O_2_) for 24 hours followed by 4 hours of re-oxygenation (95% air and 5% CO_2_).

HK-2 and RPTC cells were transfected with miR-124 mimic, inhibitor, and PARP1-shRNA, which were designed and synthesized by GenePharma corporation (Suzhou, China). Cells were incubated until the cells were 60-80% confluent, miR-124 mimic and inhibitor with final concentrations of 50 nM, PARP1 shRNA plasmid with a mass of 2.5 μg, were transfected into HK-2 and RPTCs using Lipofectamine™ RNAiMAX (Life Technologies) according to the manufacturer's instructions. The cells were collected after 48 hours.

### Animals and IRI model

C57BL/6 wild-type mice were purchased and housed in the Center of Experimental Animals of Wuhan University. All animal protocols were approved by the Animal Care and Use Committee of Renmin Hospital of Wuhan University. Mice were randomly divided into four groups (n = 8/group): pre-NC, miR-124 (agomir), anti-NC, anti-miR-124 (antagomir), all mice received a tail vein injection with 10 mg/kg miR-124 agomir and antagomir (Genepharma Shanghai, China) for 10 days (3 times a day). Additionally, PARP1 inhibitor Veliparib (Selleck Chemicals, TX, USA) was administered during renal ischemia-reperfusion injury, mice were given veliparib by gavage at a dose of 50 mg/kg/d for 2 weeks.

To induce mouse renal IRI *in vivo*, all mice (male, 8-10 weeks old, weighing 20-25 g) were abdominally anesthetized with phenobarbital sodium (60 mg/kg) firstly, then bilateral renal pedicels were clamped to induce 30 min of ischemia and 48 hours of reperfusion, the same procedures were conducted in the sham group without renal pedicle clamping.

### Cell Proliferation Assay

HK-2 cells were seeded into 96-well plates and added with Cell Counting Kit (CCK-8.10 μl/well; Dojindo, Japan), and then incubated for 1 h, The absorbance at 450 nm was finally measured using a microplate reader (Bio-Rad, CA, USA).

### Flow Cytometry

HK-2 cells were harvested and then stained with Annexin V-fluorescein isothiocyanate (FITC) for 5 min and propidium iodide (PI) (BD Biosciences, Franklin Lakes, New Jersey, USA) for 15 min in the dark. All the stained cells were analyzed by flow cytometry (FACS Calibur; BD Biosciences, Franklin Lakes, New Jersey, USA).

### Dual-luciferase reporter assay

According to the predicted binding sites of miR-124 and PARP1 by RNAhybrid 2.1 and RNA structure software, wild-type and mutate PARP1-3′UTR plasmids were designed and constructed into a pmirGlO Dual-luciferase miRNA Target Expression Vector by Promega (Madison, WI, USA). Luciferase plasmid pmirGlO-PARP1-3′UTR (wild-type/mutant) and miR-124 mimic were co-transfected into cells using Lipofectamine 2000. Luciferase assays were performed for testing the luciferase activity after transfection 48 h with the Dual-Luciferase reporter system (Promega, Madison, WI, USA).

### Serum creatinine (sCr) and blood urea nitrogen (BUN) assay

Blood was collected and immediately centrifuged at 3000 g for 10 mins at 4°C to obtain plasma for the analysis. The concentrations of sCr and BUN were measured with a fully automatic biochemical detector at the department of clinical laboratory of Renmin Hospital of Wuhan University.

### RNA extraction and real-time PCR

Total RNA was extracted from cells and tissues with Trizol reagent (Life Technologies, CA, USA) and quantified by NanoDropTM 3000 Spectrophotometer (Thermo Fisher Scientific, Waltham, USA). RevertAid First Strand cDNA Synthesis Kit (Thermo Fisher Scientific, Waltham, USA) was used for reverse transcription from RNA into cDNA, MonAmp™ Fast SYBR^®^ Green qPCR Mix (Monad Biotech Corporation, Wuhan, China) was used in the next step of Real-Time PCR analysis to test the expression levels of miR-124 and PARP1 mRNAs, the Ct values of them were calculated to compare the expressions by relative quantification (2^-ΔΔCt^) method.

### Immunohistochemistry (IHC) assay

Paraffin-embedded 4μm sections of kidney tissue were prepared, then deparaffinized and rehydrated, the antigen was exposed for 15 minutes at 100°C in sodium citrate and then incubated with 3% H_2_O_2_ at 37°C for 10 minutes. Lastly, the sections were incubated overnight at 4°C with primary antibodies against RIP1 (1:50; mouse, Santa Cruz Biotechnology, Dallas, TX, USA), RIP3 (1:100, rabbit, Novus Biologicals, Colorado, USA), TNFα (1:100, rabbit, Affinity Biosciences, Suzhou, China), Caspase8 (1:200, mouse, Proteintech, Wuhan, China) and PARP1 (1:200, mouse, Proteintech, Wuhan, China) overnight. After incubation with biotin-labeled secondary antibody for 1 h at room temperature, all the stained slides were viewed under an Olympus BX50 microscope (Olympus, Tokyo, Japan).

### Western blot analysis

Total protein of cells and renal tissues were lysed using ice-cold RIPA buffer and then supplemented with protease inhibitors and phosphatase inhibitors and centrifuged at 15, 000 g 4°C for 15 min. All membranes were incubated with primary antibodies as follows: PARP1 (1:500, rabbit, Abcam, Cambridge, UK), RIP1 (1:1000; rabbit, Abcam, Cambridge, UK), RIP3 (1:1000, rabbit, Abcam, Cambridge, UK), TNFα (1:1000, rabbit, Proteintech, Wuhan, China), Caspase8 (1:500, rabbit, Abcam, Cambridge, UK), γH2AX (1:1000; rabbit, Abcam, Cambridge, UK), RAD51 (1:1000; rabbit, Abcam, Cambridge, UK), IL-6(1:1000; rabbit, Abcam, Cambridge, UK), NFκB (1:1000; rabbit, Abcam, Cambridge, UK), GAPDH (1:10000, rabbit, Proteintech, Chicago, USA), Then they were incubated with appropriate correlated HRP conjugated secondary antibody at room temperature for 2 hours. Immunoblots were visualized by enhanced chemiluminescence apparatus (Bio-Rad, Hercules, USA). The relative integrated density values (IDVs) were calculated by Fluorchem 2.0 software with GAPDH as an internal control.

### H&E (Hematoxylin and eosin) staining

Kidney tissues were fixed in 4% paraformaldehyde and embedded in paraffin and cut into 4 µm, and stained using Hematoxylin and eosin to assess the renal tubular injury and inflammation. The histological features were observed and imaged with light microscope (Olympus, Tokyo, Japan).

### Fluorescence *in situ* hybridization

Mice kidney tissues were fixed with 4% paraformaldehyde overnight, 6 µm cryosections were treated with 1mg/ml proteinase K for 10 minutes. According to the manufacturer's instructions of Fluorescent *In Situ* Hybridization (FISH) Kit (RiboBio, Guangzhou, China), the slides were incubated with prehybridization solution at 70°C for 1 hour and then added with the mmu-miR-124-cy3 probe, heated at 65°C for 5 minutes and cooled on ice with the hybridization mixture at 37°C overnight. The sections were stained with DAPI (Invitrogen, Waltham, MA, USA) for nuclear staining after washing with SSC and PBS. All fluorescence images were captured using fluorescence microscope (Olympus, Tokyo, Japan).

### Statistical analysis

All experimental data were manifested as mean ± SD. GraphPad Prism v5.01 (GraphPad, CA, USA) was applied for statistical analysis. The date was expressed as mean ± SD. Statistical analysis was conducted using a Student's t-test or one-way ANOVA, *p* values < 0.05 were considered to be statistically significant.

## Results

### MiR-124 is suppressed in ischemic tissues and hypoxic renal tubular cells

**Figure 1:**
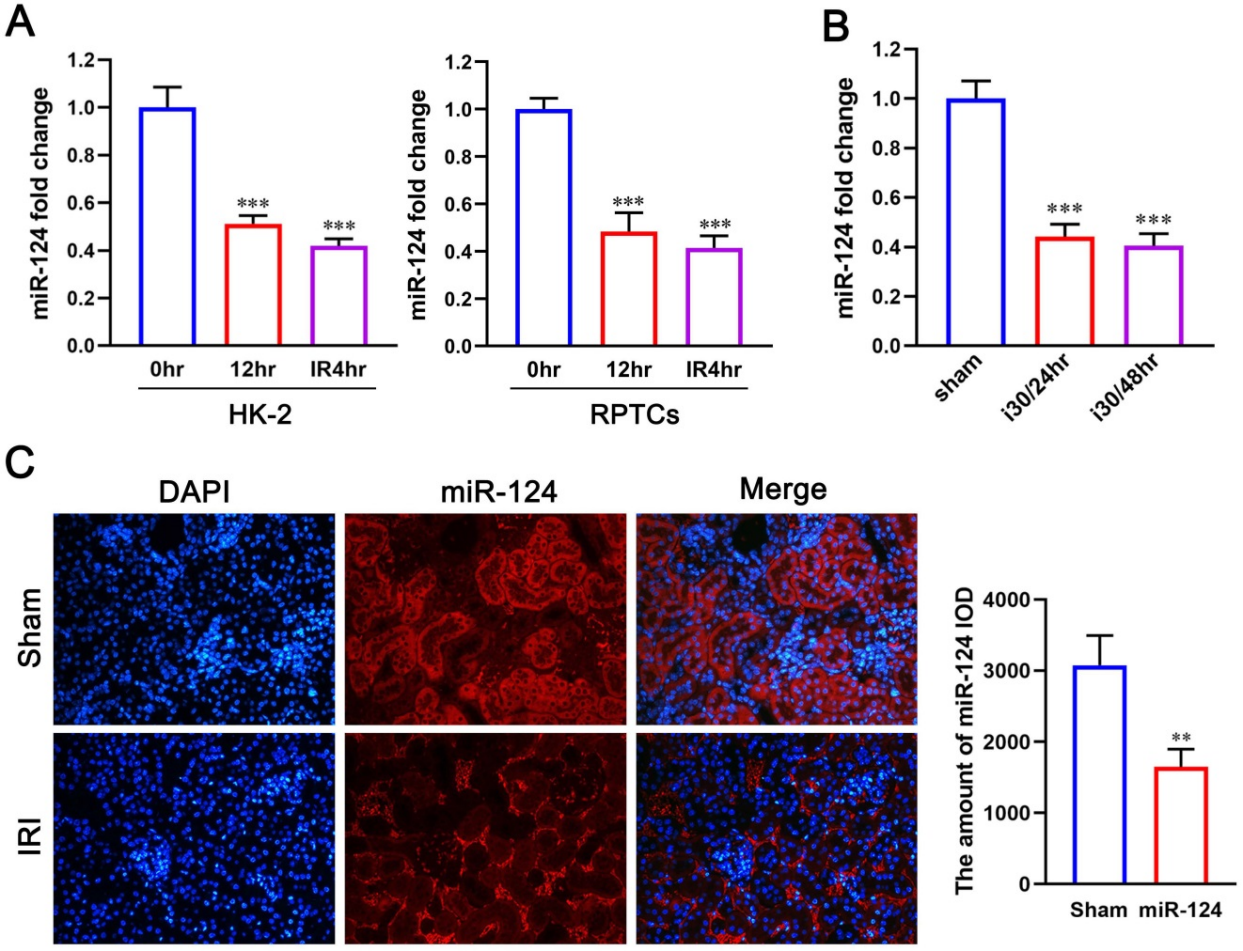
The miR-124 expression is reduced in ischemic and hypoxic renal tubular cells and mouse tissues. **(A)** MiR-124 level was examined by qPCR in HK-2 and RPTCs. **(B)** MiR-124 in mouse kidney tissues was detected by real-time qPCR. **(C)** MiR-124 was detected by fluorescence *in situ* hybridization in kidney tissues of mice. **P<0.01 ***P<0.001 compared with control or sham.

We first explored the expression of miR-124 in hypoxic renal proximal tubular epithelial cells and mouse renal tissue with ischemia-reperfusion injury by quantitative reverse transcriptase PCR. Compared with control, miR-124 decreased when suffered hypoxic incubation 24h and re-oxygenation 4h in HK-2 and RPTCs (Fig. 1A). Interestingly, miR-124 was also silenced after 30 minutes of ischemia and 24 or 48 hours of reperfusion in mouse kidney tissues (Fig. 1B). We further conducted fluorescence *in situ* hybridization (FISH) to localize miR-124 in kidneys after ischemia-reperfusion injury. The FISH showed that miR-124 was silenced in most renal tubules of ischemia-reperfusion tissues (Fig. 1C). Besides, the interstitial signals of miR-124 that appeared in interstitial staining did not change significantly after ischemia-reperfusion injury. It was suggested that miR-124 was mainly silenced in renal tubular cells.

### MiR-124 promotes cell viability and renal function *in vivo* and *in vitro*

**Figure 2:**
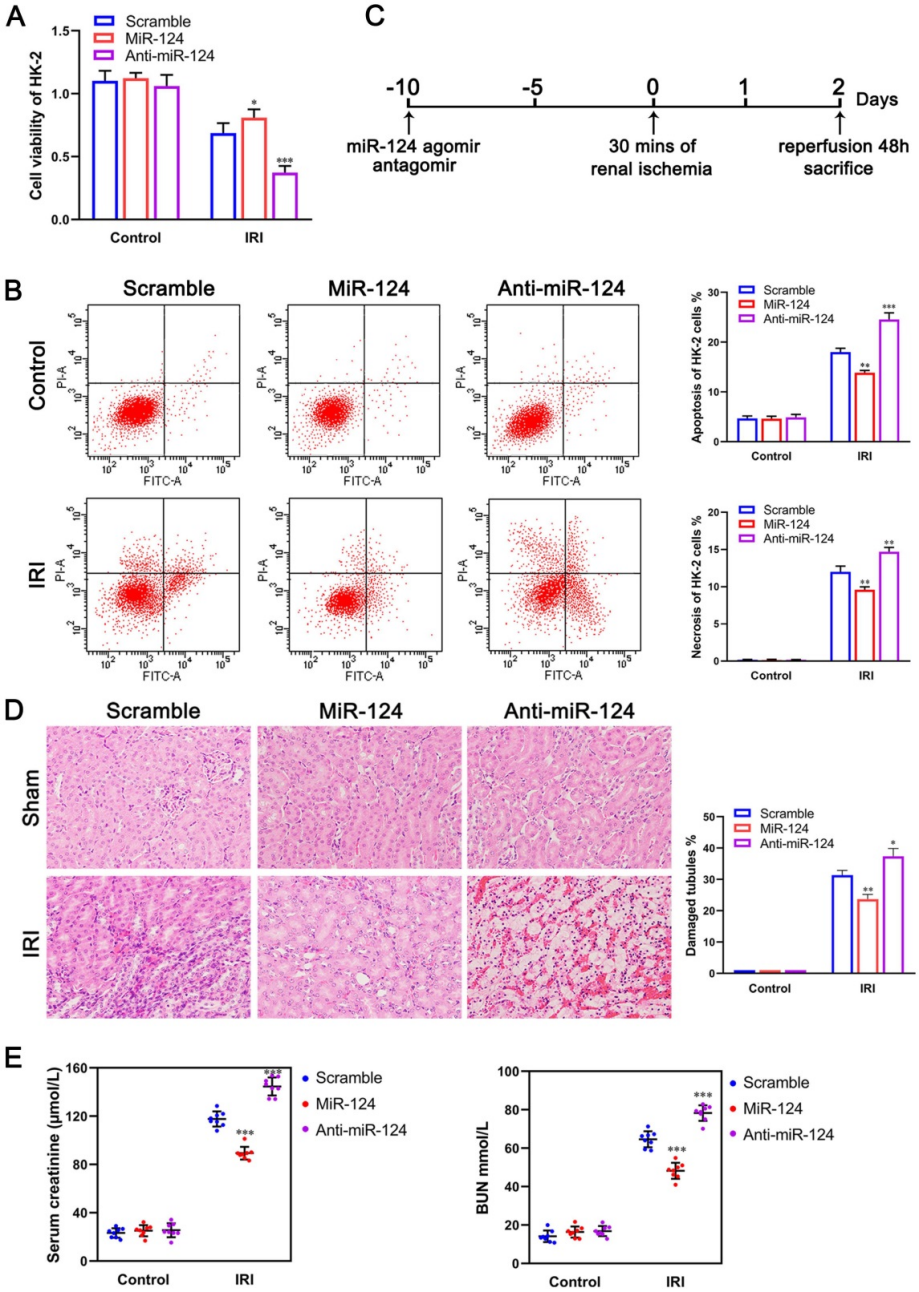
MiR-124 protects HK-2 cell and renal function *in vivo* and *in vitro*. **(A)** Effect of miR-124 on cell proliferation of HK-2 cells. **(B)** Effect of miR-124 on cell apoptosis and necrosis of HK-2 cells. **(C)** Schematics showing the timeline of mouse administration and kidney ischemia-reperfusion injury. **(D)** Representative micrographs showing H&E staining of mice with miR-124 or anti-miR-124. **(E)** Graph showing BUN and Serum creatinine level in mice with miR-124 or anti-miR-124 based on IRI. *P<0.05 **P<0.01 ***P<0.001 compared with IRI/scramble.

To examine the role of miR-124 in kidney ischemia-reperfusion injury, we transfected HK-2 cells with miR-124 mimic and inhibitor in control and ischemia-reperfusion injury cells respectively, CCK-8 assay showed that the ability of cell proliferation has no significant difference among the control groups, however, in the ischemia-reperfusion injury (IRI) group, compared to the scramble, overexpression of miR-124 could promote the cell viability of HK-2 cells while knockdown of miR-124 inhibited the cell viability under hypoxia/re-oxygenation condition (Fig. 2A). We further explored the effect of miR-124 on apoptosis and necrosis function by cell flow cytometry. The flow cytometry assay showed that miR-124 inhibited the apoptosis and necrosis significantly while knockdown miR-124 induced the apoptosis and necrosis when HK-2 cells occurred ischemia-reperfusion injury (Fig. 2B). These results revealed that miR-124 promoted cell viability and inhibited apoptosis and necrosis of HK-2 cells under hypoxia/re-oxygenation condition, therefore protecting from renal IRI.

Mice were injected with miR-124 agomir and antagomir for 10 days and then subjected to 30 minutes of bilateral renal ischemia with reperfusion 48 hours (Fig. 2C) to examine miR-124 function *in vivo*. H&E staining showed that no injury and inflammatory cell infiltration among the 3 groups regardless of miR-124. However, compared with the scramble/IRI group, overexpressed miR-124 markedly attenuated tubular injury and inflammation while anti-miR-124 aggravated tubular injury and inflammation based on IRI (Fig. 2D). We then monitored the renal function of the mice by measuring sCr and BUN. Compared with scramble/IRI mice, miR-124 treated mice showed a lower sCr and BUN after renal ischemia while anti-miR-124 treated mice represented evidently higher sCr and BUN at 48 hours of reperfusion (Fig. 2E).

### Verification of PARP1 as miR-124 target

**Figure 3:**
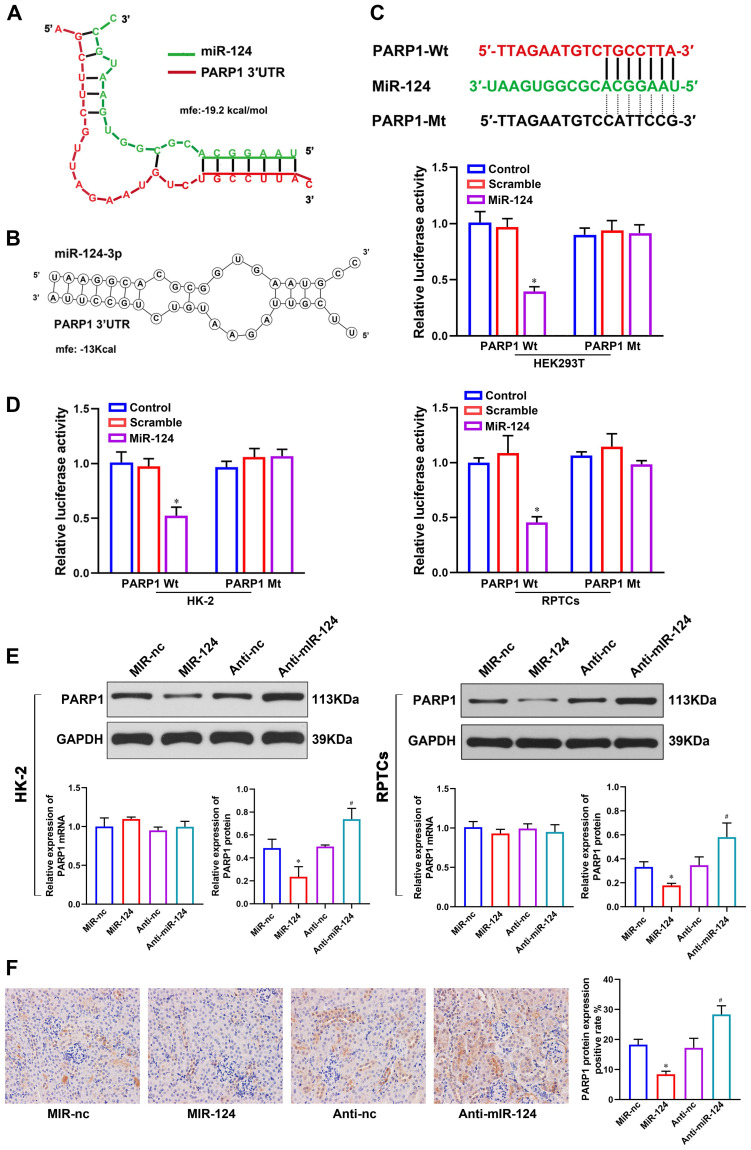
MiR-124 targets the 3′UTR of PARP1 for post-transcriptional regulation. **(A)** The predicted miR-124 binding site of PARP1 by RNAhybrid 2.1. **(B)** The predicted miR-124 binding site of PARP1 by RNA structure. **(C)** PARP1 3′UTR wild type and the designed mutant sequence (PARP1 3′UTR-Mt) are indicated. **(D)** Dual-luciferase reporter assay was conducted in HEK 293T, HK-2 and RPTCs. *P<0.05 compared with PARP1-Wt/Scramble. **(E)** PARP1 protein and mRNA expression regulated by miR-124 in HK-2 and RPTCs. **(F)** Representative micrographs showing IHC staining of PARP1 protein in mouse renal tissues. *P<0.05 compared with MiR-nc, ^#^P<0.05 compared with Anti-nc.

To better understand the mechanism of miR-124 in renal ischemia-reperfusion injury, we used RNAhybrid 2.1 (Fig. 3A) and RNA structure (Fig. 3B) software to predict the target site of miR-124 and found PARP1 3′UTR site is much outstanding. To demonstrate whether miR-124 directly targets PARP1, we designed the PARP1 3′UTR-Wild type (Wt) and Mutant (Mt) plasmids (Fig. 3C) and dual-luciferase reporter assay were performed in HEK 293T, HK-2 and RPTCs. The results showed that the relative luciferase activity only in the group co-transfected with miR-124 mimic and PARP1 3′UTR-Wt was remarkably reduced, whereas no significant differences were detected after co-transfection with the miR-124 mimic and PARP1 3′UTR-Mt in HEK 293T, HK-2 and RPTC cells (Fig. 3D). Collectively, these studies demonstrated miR-124 directly binds with PARP1 3′UTR.

To demonstrate the miR-124 regulatory mechanism of PARP1 in renal IRI, we detected the PARP1 mRNA and protein expression by overexpressing or silencing miR-124 in HK-2 and RPTC cells. The western blot showed that cells transfected with miR-124 mimic were higher levels of PARP1 protein compared to the control cells, conversely, anti-miR-124 led to a significant down expression of PARP1 protein levels both in HK-2 and RPTC cells (Fig. 3E). However, the qPCR analysis showed PARP1 mRNA had no changes when miR-124 was knockdown or overexpressed both in HK-2 and RPTC cells (Fig. 3E). Additionally, we also verified miR-124 regulation with PARP1 in mice, the IHC results showed PARP1 protein with an injection of miR-124 agamir mouse was higher than the miR-NC group, and PARP1 protein was inhibited by miR-124 antagamir in mouse renal tissues (Fig. 3F). Collectively, these results demonstrated miR-124 negatively regulated PARP1 by the post-transcriptional mechanism *in vivo* and *in vitro*.

### PARP1 inhibitor attenuates renal ischemia-reperfusion injury

**Figure 4:**
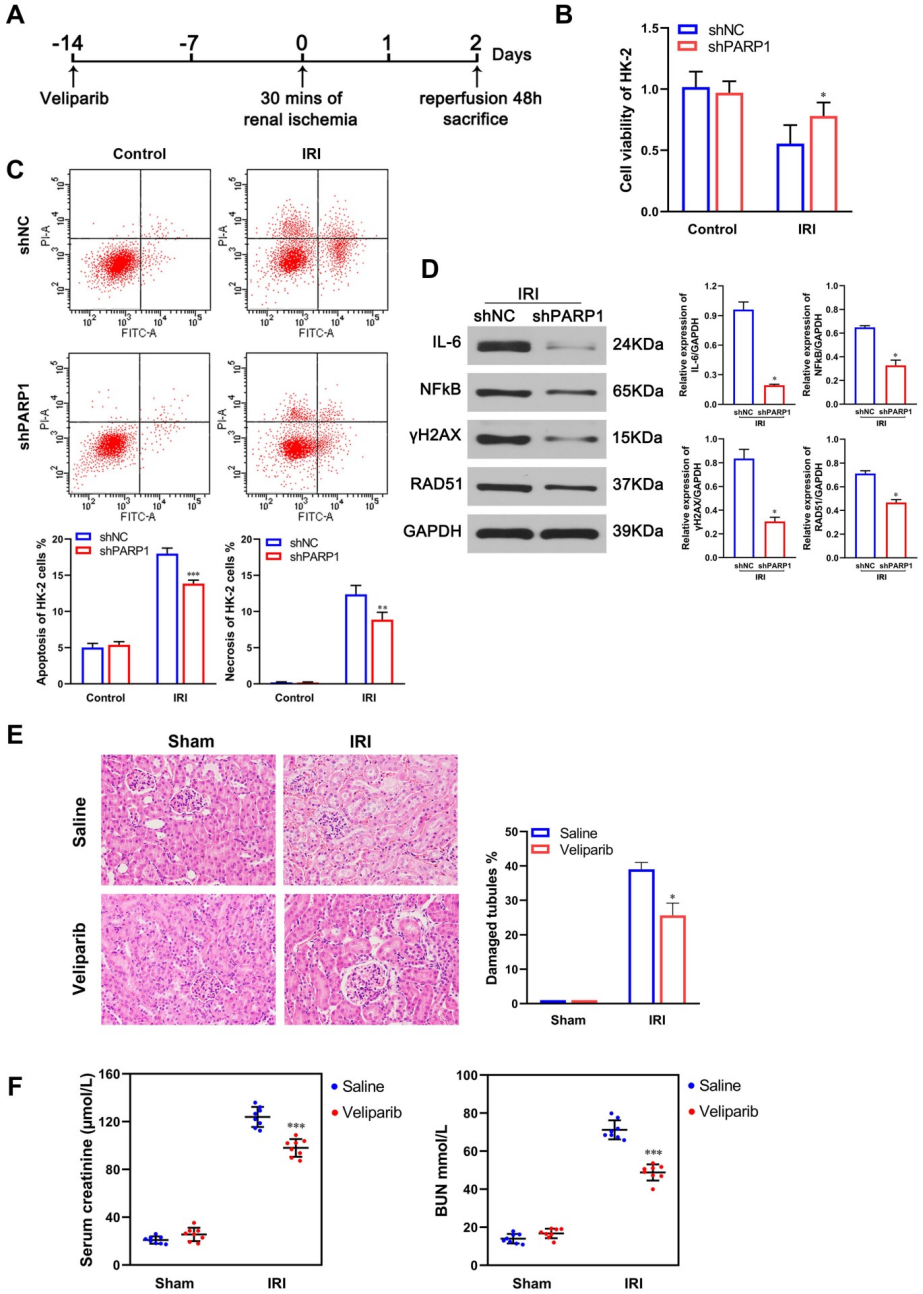
PARP1 inhibitor attenuates renal ischemic reperfusion injury. **(A)** Schematics showing the timeline of mouse administration and kidney ischemia-reperfusion injury; **(B)** Effect of shPARP1 on cell proliferation of HK-2 cells. **(C)** Effect of shPARP1 on cell apoptosis and necrosis of HK-2 cells. **(D)** Protein expressions of DNA damage and inflammatory cytokines marker proteins in HK-2 cells. *P<0.05 **P<0.01 ***P<0.001 compared with IRI/shNC. **(E)** Representative micrographs showing H&E staining of mice with veliparib. **(F)** Graph showing BUN and Serum creatinine level in mice with veliparib. *P<0.05 ***P<0.001 compared with IRI/Saline.

We demonstrated PARP1 was a target of miR-124, and whether PARP1 played a role in the protective effects of miR-124 in ischemia-reperfusion injury of HK-2 cells or mouse renal tissues. Thus, we transfected HK-2 cells with PARP1 shRNA, and fed PARP1 inhibitor veliparib by gavage in mice for 2 weeks (Fig. 4A). There were no significant differences in cell viability measured by CCK-8 assay (Fig. 4B), apoptosis and necrosis were also no different when knockdown PARP1 under normal culture conditions (Fig. 4C). However, upon the ischemia-reperfusion treatment, shPARP1 promoted cell viability, inhibited the apoptosis and necrosis significantly in HK-2 cells (Fig. 4B, C). As renal ischemia-reperfusion injury contained many complex pathological processes, such as inflammation, DNA damage and hypoxia injury, PARP1 was a DNA damage repair protein, so we detected the expression of DNA damage marker protein γH2AX and RAD51 when inhibited PARP1 in renal ischemia-reperfusion injury. It was found that γH2AX and RAD51 expressions decreased significantly in shPARP1 compared with the shNC group upon the ischemia-reperfusion injury (Fig. 4D), Meanwhile, we also detected several inflammatory cytokines IL-6 and NFκB, and the result showed that the expressions of IL-6 and NFκB were also decreased significantly after knockdown PARP1 in the ischemia-reperfusion injury. The above results showed that the inhibition of PARP1 increased cell viability, decreased cell apoptosis and necrosis, decreased DNA injury and inflammatory reaction, and greatly alleviated the renal ischemia-reperfusion injury in HK-2 cells. Furthermore, the PARP1 inhibitor veliparib attenuated tubular injury and inflammatory infiltration based on IRI by H&E staining in mice (Fig. 4E). We then measured the mouse renal sCr and BUN, compared with scramble/IRI group, mice treated veliparib consistently showed a lower sCr and BUN after renal IRI (Fig. 4F). All the results indicated that PARP1 inhibitor greatly alleviated the renal ischemia-reperfusion injury *in vivo* and *in vitro*.

### PARP1 mediates miR-124 protection of renal ischemia-reperfusion injury

**Figure 5:**
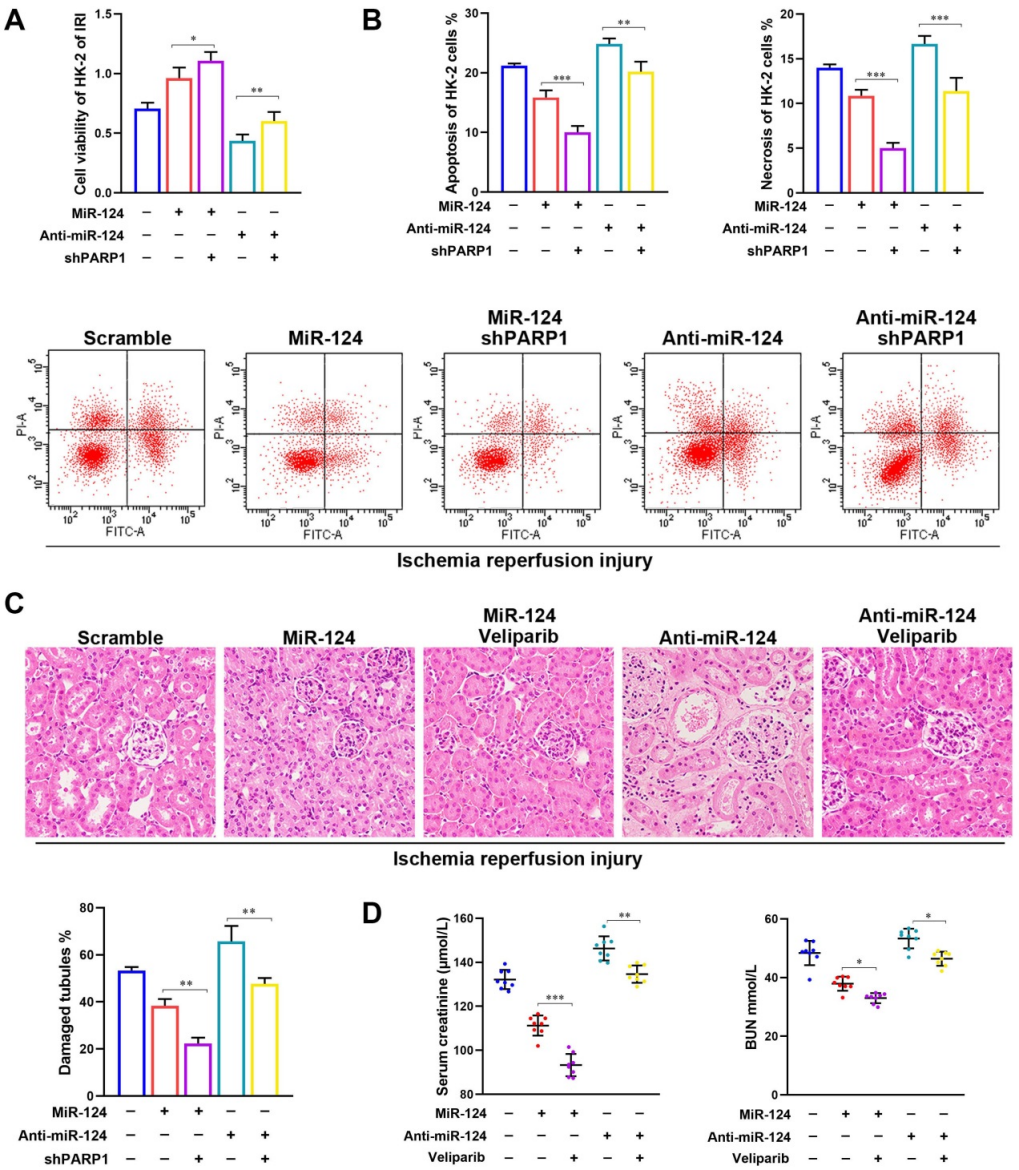
PARP1 mediates miR-124 protection of renal ischemia-reperfusion injury. **(A)** Effect of miR-124 and shPARP1 on cell proliferation of HK-2 cells. **(B)** Effect of miR-124 and shPARP1 on cell apoptosis of HK-2 cells. **(C)** Representative micrographs showing H&E staining of mice with miR-124 or anti-miR-124. **(D)** Graph showing BUN and Serum creatinine level in mice with miR-124 or anti-miR-124 based on IRI. *P<0.05 **P<0.01 ***P<0.001 compared with the corresponding group.

To determine whether the protect IRI effects of miR-124 were mediated by PARP1, the shPARP1 and inhibitor veliparib were used to rescue the protection of miR-124 in HK-2 cells and mouse kidney with ischemia-reperfusion injury. As shown in Figure 5A and 5B, based on HK-2 cell ischemia-reperfusion treatment, miR-124 promoted the cell viability and reduced the apoptosis and necrosis, on the contrary, anti-miR-124 inhibited cell viability and induced the apoptosis and necrosis, when compared with the renal ischemia-reperfusion injury, shPARP1 not only strengthened the miR-124 protection of HK-2, but also reversed the exacerbating function of anti-miR-124 on HK-2 cells viability, apoptosis, and necrosis under IRI. Furthermore, H&E staining showed the PARP1 inhibitor veliparib owned synergistic effect with miR-124 based on IRI in mouse renal tissue and rescued the injury of anti-miR-124 (Fig. 5C). Mice with veliparib consistently showed a much lower sCr and BUN with miR-124 after renal ischemia and rescued the levers of sCr and BUN with anti-miR-124 (Fig. 4D). These results indicated that the protection of miR-124 in renal IRI also be regulated by PARP1 in a negative feedback pathway *in vivo* and *in vitro*.

### MiR-124 negatively regulates PARP1 to alleviate IRI by inhibiting TNFα/RIP1/RIP3 pathway

**Figure 6:**
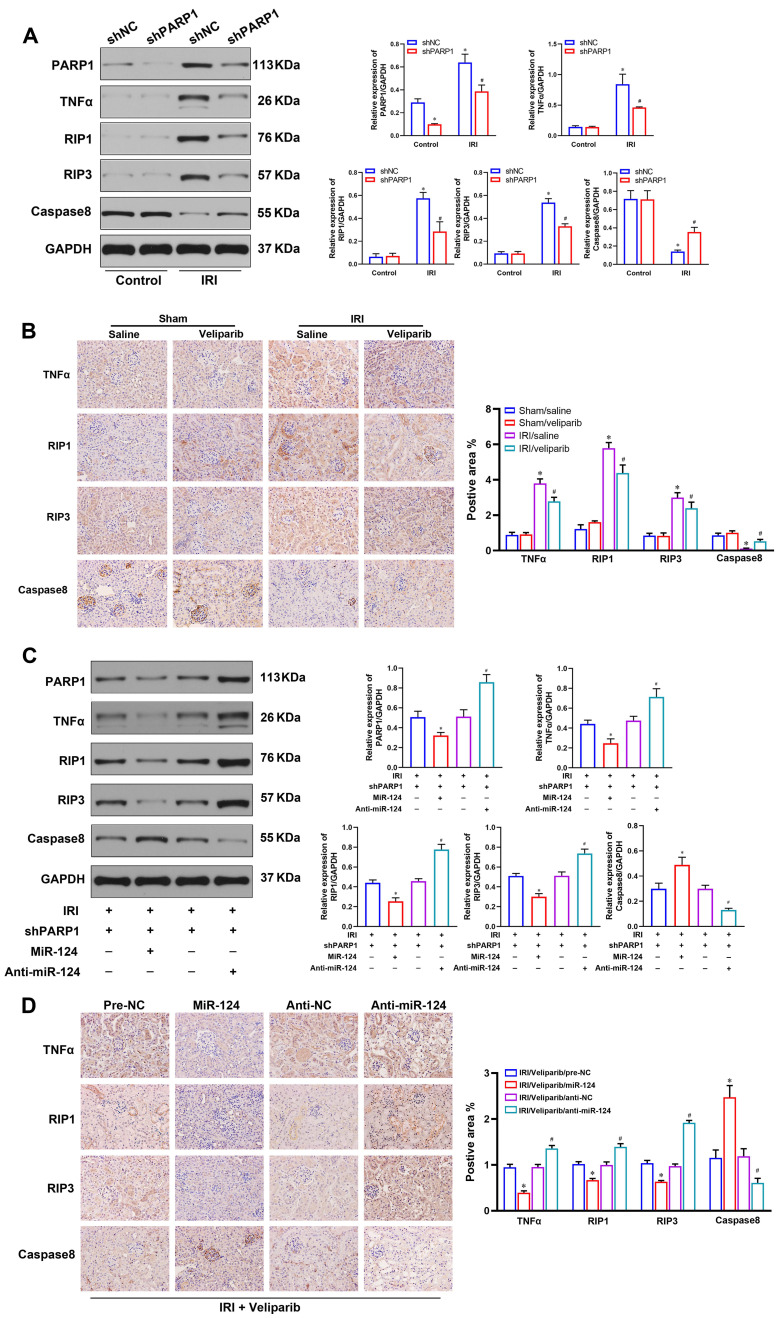
PARP1 inhibitor attenuates renal ischemia-reperfusion injury. **(A)** Protein expression of TNFα, Caspase8 and RIP1/RIP3 pathway by western blot in HK-2 cells. **(B)** Representative micrographs showing IHC staining of TNFα, Caspase8 RIP1 and RIP3 proteins in mouse renal tissues. *P<0.05 compared with Sham/Saline, ^#^P<0.05 compared with IRI/Saline. **(C)** Protein expression of TNFα, Caspase8 and RIP1/RIP3 pathway by western blot in HK-2 cells under ischemia-reperfusion injury. **(D)** Representative micrographs showing IHC staining of TNFα, Caspase8, RIP1 and RIP3 proteins in mouse renal ischemia-reperfusion injury tissues. *P<0.05 compared with IRI/veliparib/pre-NC, ^#^P<0.05 compared with IRI/veliparib/anti-NC.

As the RIP1/RIP3 pathway is a significant signaling pathway which is responsible for regulating cell viability and necroptosis in renal IRI 22, TNFα is a classic inflammatory cytokine, but a familiar inducer of necroptosis in some pathological process, particularly in the disease containing inflammation and necrosis 23. Inhibition of caspase8 can also induce necroptosis of renal tubular epithelial cells 17. We further explored the relationship among PARP1, TNFα, Caspase8 and RIP1/RIP3 pathway in HK-2 cells and mouse renal tissues. The western blot showed the expressions of TNFα, Caspase8, RIP1, RIP3 were not induced in normal condition, but TNFα, RIP1 and RIP3 were activated, and Caspase8 was inhibited in renal IRI. While, shPARP1 could inhibit TNFα, RIP1 and RIP3 expressions and increase Caspase8 under ischemia-reperfusion injury in HK-2 cells (Fig. 6A). The IHC consistently showed TNFα, Caspase8 RIP1 and RIP3 proteins had no changes in sham mice, but veliparib suppressed the protein of TNFα, RIP1 and RIP3, and activated Caspase8 in renal IRI tissues (Fig. 6B).

Moreover, to study the effect of miR-124 on TNFα, Caspase8, RIP1 and RIP3 expressions with PARP1 inhibitor transfected HK-2 cells and mouse tissues under IRI treatment, we pre-transfected cells with shPARP1 24 hours followed by ischemia-reperfusion treatment for 24 hours. The result showed that miR-124 mimic decreased TNFα, RIP1, RIP3, and activated Caspase8 by western blot, whereas anti-miR-124 increased TNFα, RIP1, RIP3, and inhibited Caspase8 protein levels under renal ischemia-reperfusion conditions (Fig. 6C). The IHC consistently showed TNFα, RIP1, RIP3 proteins were inhibited, Caspase8 was increased by miR-124 mimic, while anti-miR-124 induced the protein of TNFα, RIP1, RIP3, and inhibited Caspase8 protein expressions in renal IRI tissues (Fig. 6D). These results revealed that miR-124 negatively regulated PARP1 to alleviate IRI by inhibiting TNFα/RIP1/RIP3 pathway *in vivo* and *in vitro*.

## Discussion

In this study, we demonstrated that miR-124 was a lower expression in renal proximal tubular epithelial cell HK-2 and mouse renal tissues of ischemia-reperfusion injury. Overexpressed miR-124 markedly attenuated tubular injury and inflammation while anti-miR-124 aggravated tubular injury and inflammation based on IRI. The further study showed that miR-124 negatively regulated PARP1 by post-transcription, and PARP1 also mediated miR-124 protection in a negative feedback pathway in renal ischemic-reperfusion injury. The mechanism study found that miR-124 targeted PARP1 3′UTR and PARP1 activated cell apoptosis and necrosis by RIP1/RIP3 pathway. Taken together, miR-124 played a role in the protection of ischemia-reperfusion injury by negatively regulated PARP1 and TNFα/RIP1/RIP3 pathway, which suggested a promising target in AKI and renal transplantation.

Renal ischemia-reperfusion injury is the main cause of acute renal injury 24 and an unavoidable problem in renal transplantation 25. Recent studies have shown that some miRNAs are extensively involved in several organs and tissues of IRI 8, 26. MiR-124 has been reported to participate extensively in the pathophysiology of ischemic stroke, functioning as a protective role against cerebral ischemia-reperfusion injury 27, 28. MiR-124-3p was significantly downregulated in both myocardial ischemia-reperfusion injury rat model and hypoxic/re-oxygenation treated human cardiac myocyte 29. Another study confirmed miR‑124 regulated inflammatory signaling via an MCP1-dependent mechanism to protect renal in septic mice 30. Our study found miR-124 was suppressed in mouse renal IRI tissues and hypoxia/re-oxygenation renal tubular cells, further study showed miR-124 mimic promoted the cell viability and inhibited apoptosis and necrosis of HK-2 cells under hypoxia/reoxygenation condition, and markedly attenuated tubular injury and inflammation of mouse renal IRI tissues. These results provided a piece of solid evidence for a miR-124 functional role in renal ischemia-reperfusion injury.

PARP1 has been found to induce renal IRI and its inhibitors could protect the kidney in some cell and animal models of renal IRI 31, 32. Necrostatin-1 increased the expression of PARP1 and inhibited RIP1/RIP3 signaling pathway, finally attenuated the renal IRI and cell necroptosis 6, 33 Moreover, another report illustrated that inhibition of PARP1 can prevent kidney dysfunction and tubular necrosis of renal IRI 19, 34. Consistent with our results, shPARP1 transfection could promote cell viability and inhibited apoptosis and necrosis significantly in HK-2 cells, the PARP1 inhibitor veliparib also attenuated tubular injury and inflammation based on IRI mouse renal. In addition, shPARP1 reduced DNA damage marker protein γH2AX and RAD51 in HK-2 cells under hypoxia/reoxygenation conditions. The inflammatory cytokines IL-6 and NFκB were also decreased significantly after knockdown PARP1 in the renal ischemia-reperfusion injury. So inhibition of PARP1 could alleviate renal ischemia-reperfusion injury by reducing the DNA damage, inflammation and cell necroptosis.

Additionally, we also found a new miRNA-PARP1 regulatory mechanism to protect renal from ischemia-reperfusion injury. Firstly, we used RNAhybrid 2.1 and RNA structure software to predict the target site of miR-124 and found the binding site with PARP1. Secondly, luciferase reporter assay has been performed both in HEK 293T cells to verify the binding site between miR-124 and PARP1 3′UTR-Wt. Lastly, western blot and IHC experiments demonstrated miR-124 negatively regulated PARP1 expression by a post-transcriptional mechanism *in vivo* and *in vitro*. Furthermore, PARP1 inhibitor also reversed the exacerbating effects of anti-miR-124 on HK-2 cells and renal tissues with IRI. These all demonstrated miR-124 bound with PARP1 3′UTR and negatively regulated the expression and functions in renal IRI.

Some studies elaborated that renal cells undergo apoptosis, necrosis, or necroptosis following IRI 35, 36. Necroptosis was induced by the activation of the death receptor complex RIP1/RIP3/MLKL on the cell membrane 22. RIP1 and RIP3 were the core proteins in the necroptosis signaling pathway. TNFα mediated necroptosis is one type of necroptosis cell death in many diseases, such as glioma cells 37 and alveolar epithelial cells 38. TNFα can also induce necroptosis of inflammatory cells by activating RIP1 and RIP3 39. Activation of RIP3 induced necroptosis of renal tubular epithelial cells, knockdown of RIP3 reduced the degree of renal injury, and inhibition of Caspase8 also induced necroptosis in renal tubular epithelial cells 17. Caspase8 suppressed RIP1-RIP3 kinase complex-dependent necroptosis that followed death receptor activation in mice 40. In our studies, both western blot and IHC showed that shPARP1 inhibited the expressions of TNFα, RIP1 and RIP3, but increased Caspase8 under ischemia-reperfusion injury status in HK-2 cells and renal tissues. So we conjectured that PARP1 could induce necroptosis by activating TNFα and downstream molecules. As we have confirmed miR-124 negatively regulated PARP1 protein in renal IRI, miR-124 mimic and inhibitor were transfected to explore the internal mechanism in renal IRI *in vivo* and *in vitro*. The result showed that anti-miR-124 significantly induced TNFα, RIP1, RIP3 expressions, and inhibited Csapase8 by western blot and IHC under renal IRI conditions. Therefore, we believed miR-124 negatively regulated PARP1 to alleviate IRI by inhibiting TNFα/RIP1/RIP3 pathway.

Unfortunately, my study did not explore the functions of TNFα in the process of necroptosis. As there were large parts of complicated biological molecules in cell death pathways, and TNFα may induce necroptosis by some other pathways. Researchers should further explore the underlying mechanism of PARP1 activating TNF receptor in apoptosis, necroptosis, and other ways of cell death in the renal IRI process.

**Figure 7:**
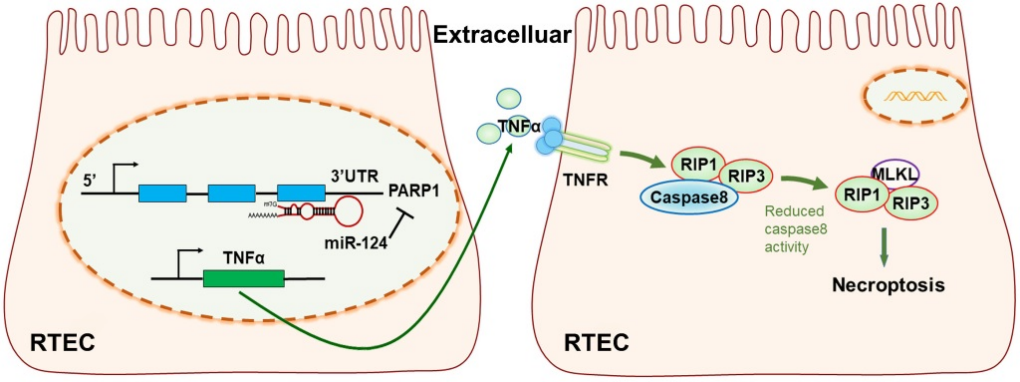
The mechanism diagram of the relationship among miR-124, PARP1 and TNFα/RIP1/RIP3 pathway in renal tubular epithelial cells (RTEC).

In conclusion, our study revealed miR-124 was suppressed in ischemic tissues and hypoxic renal tubular epithelial cells (RTEC), miR-124 promoted cell viability and renal function *in vivo* and *in vitro* by targeting the 3′UTR of PARP1 for post-transcriptional regulation. Anti-miR-124 regulated PARP1 to induce TNFα activity, and TNFα further inhibited Caspae8 and activated RIP1/RIP3 pathway to deteriorate renal cell necroptosis, leading to the exacerbation of the renal ischemia-reperfusion injury and related functions (Fig. 7). Therefore, miR-124 could negatively regulate PARP1 to alleviate renal ischemia-reperfusion injury by inhibiting TNFα/RIP1/RIP3 pathway, miR-124 may be exploited as a novel therapeutic agent to attenuate IRI in renal acute injury and kidney transplantation.
